# The toxic effects of electronic cigarette aerosol and cigarette smoke on cardiovascular, gastrointestinal and renal systems in mice

**DOI:** 10.1038/s41598-023-39201-7

**Published:** 2023-07-31

**Authors:** Aijing Xu, Kun Duan, Wanchun Yang, Guodong Feng, Zehong Wu, Xingtao Jiang, Min Li, Peiqing Liu, Jianwen Chen

**Affiliations:** 1 Department of Pharmacology and Toxicology, School of Pharmaceutical Sciences, Sun Yat-Sen University, Guangzhou, 510006 Guangdong China; 2 National and Local Joint Engineering Laboratory of Druggability and New Drugs Evaluation, Guangdong Engineering Laboratory of Druggability and New Drug Evaluation, School of Pharmaceutical Sciences, Sun Yat-Sen University, Guangzhou, 510006 China; 3 RELX Science Center, Shenzhen RELX Tech. Co., Ltd., Shenzhen, 518101 China

**Keywords:** Physiology, Cardiology, Gastroenterology

## Abstract

Electronic cigarette (EC) has been suggested to be less harmful than cigarette smoking, but the research on the full extent of their harm reduction potential is still lacking. This study aimed to evaluate the influence of EC aerosol and cigarette smoke (CS) on cardiovascular, gastrointestinal, and renal functions in mice after prolonged exposure. Forty-eight C57BL/6J male mice were randomly grouped and then exposed to fresh air (control), mung bean-flavored EC aerosol with low and high dose (EC1L, 6 mg/kg; EC1H, 12 mg/kg), watermelon-flavored EC aerosol with low and high dose (EC2L, 6 mg/kg; EC2H, 12 mg/kg), and finally a cigarette smoke (CS, 6 mg/kg), respectively. After 10 weeks of exposure, the heart rate increased for both the EC and CS groups, and the effect of CS on blood oxygen saturation was significantly higher than that of the EC group (P < 0.01). Proteomic analysis of the heart tissue showed that the overlapped differential expression protein from the EC and CS exposures was Crip2. For the gastrointestinal system, oral mucosa was significantly damaged in CS group. Compare with CS, EC had significantly fewer negative effects on most of the indictors which focused on in this study.

## Introduction

Smoking is a major health threat to both smokers and bystanders, and more than 1 billion smokers worldwide are habitual tobacco users and the number is still increasing^1–3^. It is well known that smoking causes many diseases, including lung cancer, cardiovascular disease, and respiratory diseases^4,5^. Cigarette smoke (CS) contains more than 9000 identified chemicals and more than 69 known human carcinogens^6^.

Electronic cigarette (EC) is a relatively new and developing nicotine inhalation system^7^, which produces an aerosolized mixture of flavoring e-liquids with/without nicotine^8^. In recent years, there have been four generations of EC devices on the market, the latest one being closed pod e-cigarette^9,10^. Despite their different shapes and sizes, all ECs function similarly and usually consist of three main components: a lithium-ion battery power source, a case that holds the e-liquid and the control circuits, and a vaporizer or heater that vaporizes the e-liquid^11,12^. Sales of EC have increased exponentially over the past decade in some markets, and EC has become a multi-billion dollar industry^13^, thus concerns are also rising about their safety^14^.

EC is considered an alternative to cigarettes, containing 9 to 450 times fewer harmful compounds than those found in conventional cigarette smoke^15^. However, this does not mean that EC has no harmful effects^16^. Studies have detected approximately 250 chemicals in EC aerosols including nicotine, fragrance, volatile organic compounds (VOCs), pyridine and carbonyl compounds^17,18^, which were reported to affect the respiratory system^19,20^, central nervous system^21^, immune system^22,23^, throat and mouth inflammation^24^. Recent studies have reported that the use of EC is related to inflammation, oxidative stress, and hemodynamic imbalance^25^. Therefore, more experimental and clinical studies on the acute and chronic effects of EC are needed to establish its safety or risk, and its potential as an auxiliary means of quitting smoking.

Studies have shown that smoking causes many diseases^26–29^. However, the effects of different exposure conditions on the main organs of the cardiovascular, gastrointestinal, and renal systems are poorly understood. The harm caused by vaping has been related to many factors, including exposure dose, exposure time, or the composition of e-liquid. Therefore, it is necessary to evaluate multiple variables involved in EC/CS exposure. Nicotine is the main addictive component of cigarettes, and its content will be different in the EC sold on the market^30^. Differences in the distribution kinetics of nicotine in EC may lead to different effects on the cardiovascular system and gastrointestinal tract, so further research is essential to determine whether the release of nicotine and its concentrations in EC produces toxicity similar to that of CS. In addition, a large number of studies have shown that certain flavoring molecules in EC aerosol can cause respiratory diseases^31–33^, but their effects on the cardiovascular and gastrointestinal systems remain unknown. Therefore, studies that evaluate specific changes in the cardiovascular and gastrointestinal systems caused by EC flavour may also be critical to determine the health risks of EC.

Therefore, in this study we included two popular EC flavors and a commercial cigarette. We used C57BL/6J male mice as the animal model to explore and compare the effects of CS and EC on the cardiovascular, gastrointestinal and renal systems. Under a set of different exposure conditions, physiological and pathological changes in the three main organs were examined. At the same time, proteomics analysis of heart tissue was carried out to explore the different toxic mechanisms of EC and CS on the heart.

## Materials and methods

### Instrument and reagents

EC1 (mung bean-flavored) and EC2 (watermelon-flavored) were manufactured by Shenzhen RELX Tech. Co., Ltd. (China). They are closed-pod type ECs with ceramic atomizers, and both e-liquids contained 3% nicotine, and had a power output of 6.5 watts. The cigarettes used in the study were a commercially available brand in China, with pack-labeled 10 mg tar and 1.0 mg nicotine per cigarette. Passive smoking animal staining systems (Beijing Huironghe Technology Co., Ltd, Model: HRM-MNE3026), an ultra-high resolution small animal ultrasound imaging system (FujiFilm VisualSonics, Canada, model: Vevo2100), a pulse oximeter (Starr Mouse Ox, USA, model: MouseOx), an optical microscope (Nikon, Japan, model: NIKON Eclipse ci) with an imaging system (Nikon, Japan, model: NIKON digital sight DS-FI2) were used. Dimethyl sulfoxide (DMSO, 1.23041.010) was purchased from Jinhuada (Guangzhou, China). The HE staining kit was purchased from Boster Biological Technology Co., Ltd. (Wuhan, China).

### Animals

Forty-eight specific pathogen-free male C57BL/6J mice, weighing approximately 16–18 g each, were purchased from Guangdong Medical Laboratory Animal Centre (Certificate No. 44007200091696, China). They were kept in a 12-h light/dark cycle with a room temperature of 22 °C and had ad libitum access to food and water. The animal experiments were performed based on the guidelines for the care and use of animals and approved by the Institutional Animal Care and Use Committee of Guangzhou Boji Medical Biotechnological Co., Ltd. All methods were carried out in accordance with ARRIVE guidelines and regulations.

### Smoke and aerosol collection and nicotine measurement

Aerosol from the EC or the mainstream smoke from CS was collected with a Cambridge filter in a whole-body chamber. The size of the chamber was 3.8 L, the flow rate of the air pump was 2.4 L/min. This smoke delivery method simulated the way a standard smoking machine produces smoke: 55 mL puff volume was released in 3 s, paused for 27 s, and released again. Smoke/aerosol was released at a rate of 2 times per minute. The total amount of smoke/aerosol released was 110 mL/min, and after 30 min the Cambridge filter was extracted with 10 mL of DMSO. The average nicotine contents of EC and CS were determined within 30 min using ultra-performance liquid chromatography (UPLC). The mobile phase was made up of 10 mM ammonium acetate (A) and 0.3 mL/min acetonitrile (B). The analytical column was an ACQUITY UPLC HSS T3 2.1 × 100 mm × 1.8 µm. The injection volume and temperature of the column were 0.5 μL and 40 °C, respectively. The following was the best gradient elution: from 0 to 6.0 min, A–B of 80:20, from 6.01 to 9.0 min, A–B of 10:90; and from 9.01 to 12.0 min, A–B of 80:20. At a wavelength of 260 nm, UV chromatograms were detected.

### Design and dosage of animal exposure

The results of UPLC experiments showed that the average concentration of nicotine was 0.1 mg/L. The nicotine doses for animal experiments were obtained after the modification of previous experimental methods^34^. For the test mice weighing an average of 0.02 kg, the ventilation volume per minute was 0.0217 L/min^35^. Based on this calculation, mice exposed to smoke for 60 min per day could achieve a nicotine dose of 6 mg/kg. Six mg/kg was used as the low dosage for EC and CS. The high dosage for EC was selected as 12 mg/kg. Thus, the mice exposed to the EC aerosol for 60 min and 120 min reached the equivalent doses of 6 mg/kg and 12 mg/kg, respectively, and mice in the CS group were exposed to smoke for 1 h per day.

### Procedure for EC and CS exposure

The forty-eight specific pathogen-free C57BL/6J male mice were randomly divided into six groups, including two groups for EC low and high dose (mung bean), two groups for EC low and high dose (watermelon), one group for CS and the control group. They exposed to fresh air (control), mung bean-flavored EC aerosol with the low and high dose (EC1L, nicotine 6 mg/kg; EC1H, nicotine 12 mg/kg), watermelon-flavored EC aerosol with the low and high dose (EC2L, nicotine 6 mg/kg; EC2H, nicotine 12 mg/kg), and finally a cigarette smoke exposure (CS, 6 mg/kg). The CS group was exposed to smoke for 1 h per day. Except for the control group, the EC1L and EC2L groups were exposed to aerosol twice a day for 30 min each, the EC1H and EC2H groups were exposed to aerosol four times a day for 30 min each. The total exposure lasted 10 weeks, 5 days a week. Smoke/aerosol was delivered in the same manner as the above collection method.

### Detection of cardiac function in animals

The ejection fraction (EF), left ventricle shortening fraction (FS), left ventricular anterior wall- diastole (LVAWd), left ventricular anterior wall-systole (LVAWs), left ventricular posterior wall- diastole (LVPWd) and left ventricular posterior wall-systole (LVPWs) of mice were detected by an ultrahigh resolution small animal ultrasound imaging system.

### Blood oxygen saturation and heart rate

After the cardiac functions, the blood oxygen saturation and heart rate of mice were measured by a Starr pulse oximeter.

### Cardiac tissue sampling and index detection

After the blood oxygen index of the animal was measured, the animal was anesthetized with 1% pentobarbital sodium (50 mg/kg), the chest and abdominal cavity were opened. The heart was removed after the blood was collected, the residual blood was removed and weighed, and the cardiac index (heart weight/body weight × 100%) was calculated. Masson staining was performed for morphologic assessment by routine fixation in 4% paraformaldehyde fluid routine.

### Oral sampling and index detection

The animals were anesthetized with 1% pentobarbital sodium (50 mg/kg), and the changes in oral mucosa were observed under cold light source with reference to the Sonis standard score. The scoring criteria were as follows: 0 points, normal mucosa; 1 mark, erythema and local vascular dilatation; 2 points, severe erythema and vascular dilatation with punctate erosion and exfoliation; 3 points, one or more ulcers, ulcer area less than 25%; 4 points, one or more ulcers, ulcer area between 25 and 50%; and 5 points, one or more ulcers, ulcer area greater than 50%. The bilateral oral buccal mucosa of the animal was cut out with a volume of approximately 0.5 cm × 0.5 cm × 0.2 cm. The tissue block was flatted, and the original shape was maintained as much as possible. Care was taken to clamp the tissue to avoid tissue deformation, and the removed mucosa was fixed in 4% paraformaldehyde solution.

### Gastrointestinal sampling and index detection

After the mice were sacrificed, the abdominal cavity was opened, and the stomach and duodenum were cut out. Any food residues were cleaned with saline, then fixed in 4% paraformaldehyde solution, and the histopathological changes were observed by staining sections.

### Liver tissue sampling and index detection

The liver was removed during sampling, and the gall bladder was cut off and weighed to calculate the liver index (liver weight/body weight × 100%). The liver tissue of the same part was cut and fixed in 4% paraformaldehyde solution. After sectioning, HE staining was used to observe the histopathological changes, and Masson staining was used to observe the fibrosis of liver cells.

### Kidney sampling and index detection

The left kidney was weighed, and the kidney index (kidney weight/body weight × 100%) was calculated. The renal capsule was removed and fixed in 4% paraformaldehyde solution. After sectioning, histopathological changes were observed by HE staining, and fibrosis was observed by Masson staining.

### Proteomics analysis

Heart tissues were obtained and immediately frozen in liquid nitrogen. Each group provided three biological replicates for proteomic analysis by Lc-Bio Technologies (Hangzhou, China). The heart tissue was processed (including protein extraction, denaturation, reduction, alkylation, trypsin digestion and TMT labeling), and then the peptide was separated by HPLC‒MS/MS, and the data were analyzed by MaxQuant Search Engine (v.1.5.2.8). The screening criteria for differential expression analysis were fold change (FC) > 1.3 and *P* value < 0.05. Bioinformatic analysis was performed using the OmicStudio tools at https://www.omicstudio.cn/tool^36^. The function of the identified proteins was analyzed using gene ontology (GO) terms.

### Statistical analysis

For all the experiments, 8 mice were studied per group unless otherwise stated. Means ± SEM were calculated, and statistical analysis was performed using GraphPad Prism version 8.00 for Windows. The count data were analyzed by the Kruskal–Wallis test with Dunn’s multiple comparisons test. The measurement data were ascertained by ordinary one-way ANOVA with Tukey’s multiple comparisons test, and statistical significance was set at *P* < 0.05.

### Ethics approval

The animal experiments were performed based on the guidelines for the care and use of animals and approved by the Institutional Animal Care and Use Committee of Guangzhou Boji Medical Biotechnological Co., Ltd. All methods were carried out in accordance with ARRIVE guidelines and regulations.

## Results

### Toxic effects of EC and CS exposure on the cardiovascular system in mice

**Figure 1:**
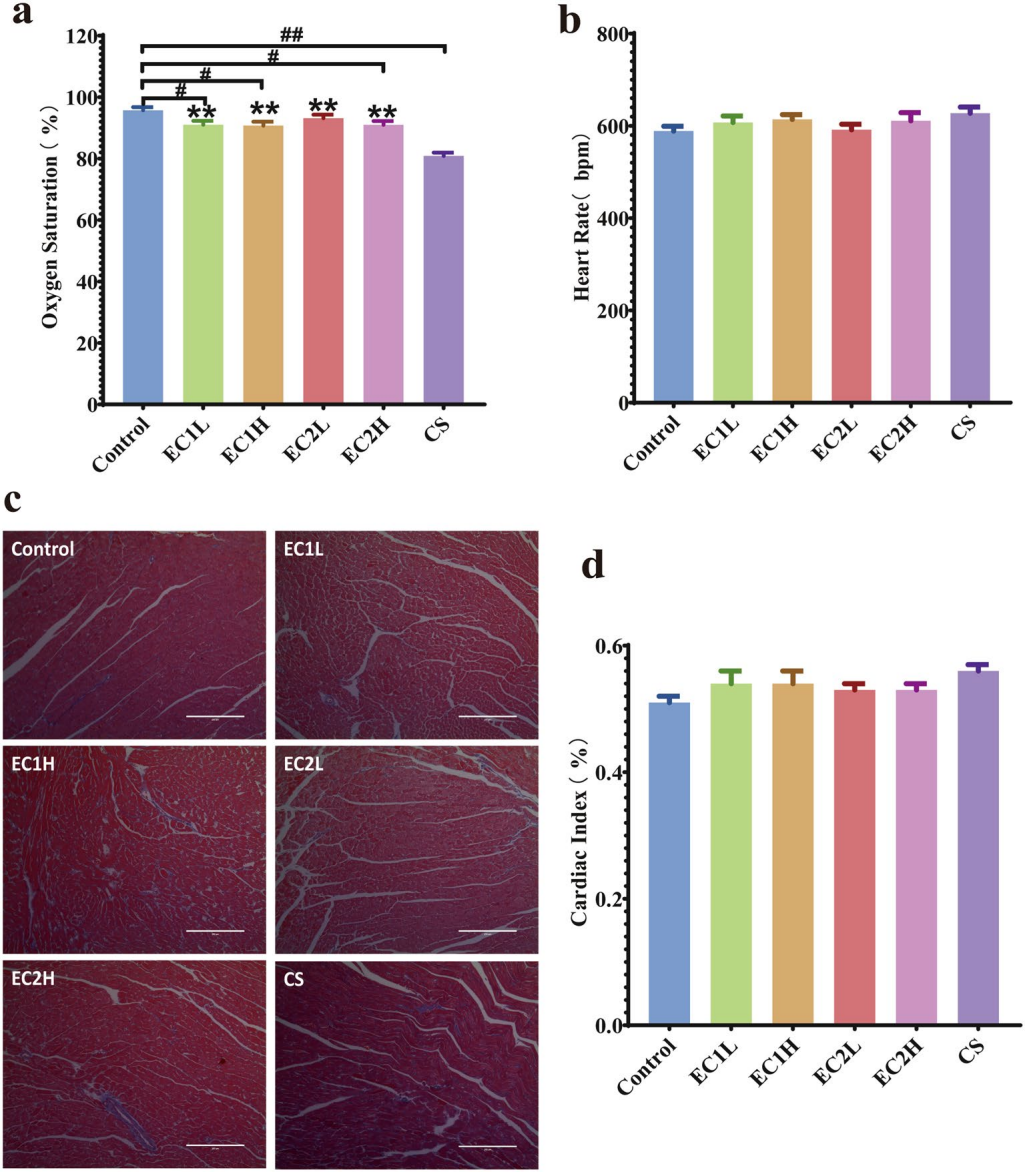
Effects of EC and CS on (**a**) blood oxygen saturation (n = 7–8); (**b**) heart rate (n = 7–8); (**c**) pathological changes in the heart (Masson, × 100) and (**d**) cardiac index (n = 7–8) in mice. Data are represented as the mean ± SEM. ^#^*P* < 0.05, ^##^*P* < 0.01 vs. Control, *P < 0.05, **P < 0.01 vs. CS.

In vivo cardiac function, the indexes EF, FS, LVAWd, LVAWs, LVPWd and LVPWs of the mice in each group showed no abnormality and no significant difference (*P* > 0.05) (Suppl. Fig. S1). Compared with the control group, 10 weeks of exposure to EC and CS had no significant changes in the basic morphology and function of the heart (Suppl. Fig. S2). As shown in Fig. 1a, compared with the control group, the blood oxygen saturation of EC1L, EC1H and EC2H decreased significantly (*P* < 0.05), while which was more pronounced in the CS group (*P* < 0.01). Compared with the CS group, the blood oxygen saturation in all EC groups increased with statistical difference (*P* < 0.01). Compared with the control group, the heart rate of mice in other five groups increased to varying degrees. Among them, the heart rate in CS increased more, but there was no significant difference among the EC groups (Fig. 1b). This suggests that CS exposure was more likely to induce a decrease in blood oxygen saturation and affect circulatory physiological function than EC exposure. Masson staining of the heart (Fig. 1c) showed that the myocardial fibers in each group were arranged orderly, the cytoplasm was rich and uniform, and there was no obvious increase in collagen fibers. Compared with the control, the cardiac index of EC and CS increased, which was most obvious in the CS (Fig. 1d), and there was no significant difference between all the other groups. The results indicated that 10 weeks of exposure to EC and CS caused no obvious pathological damage to the heart.

### Proteomics analysis of heart tissue in mice exposed to EC and CS

**Figure 2:**
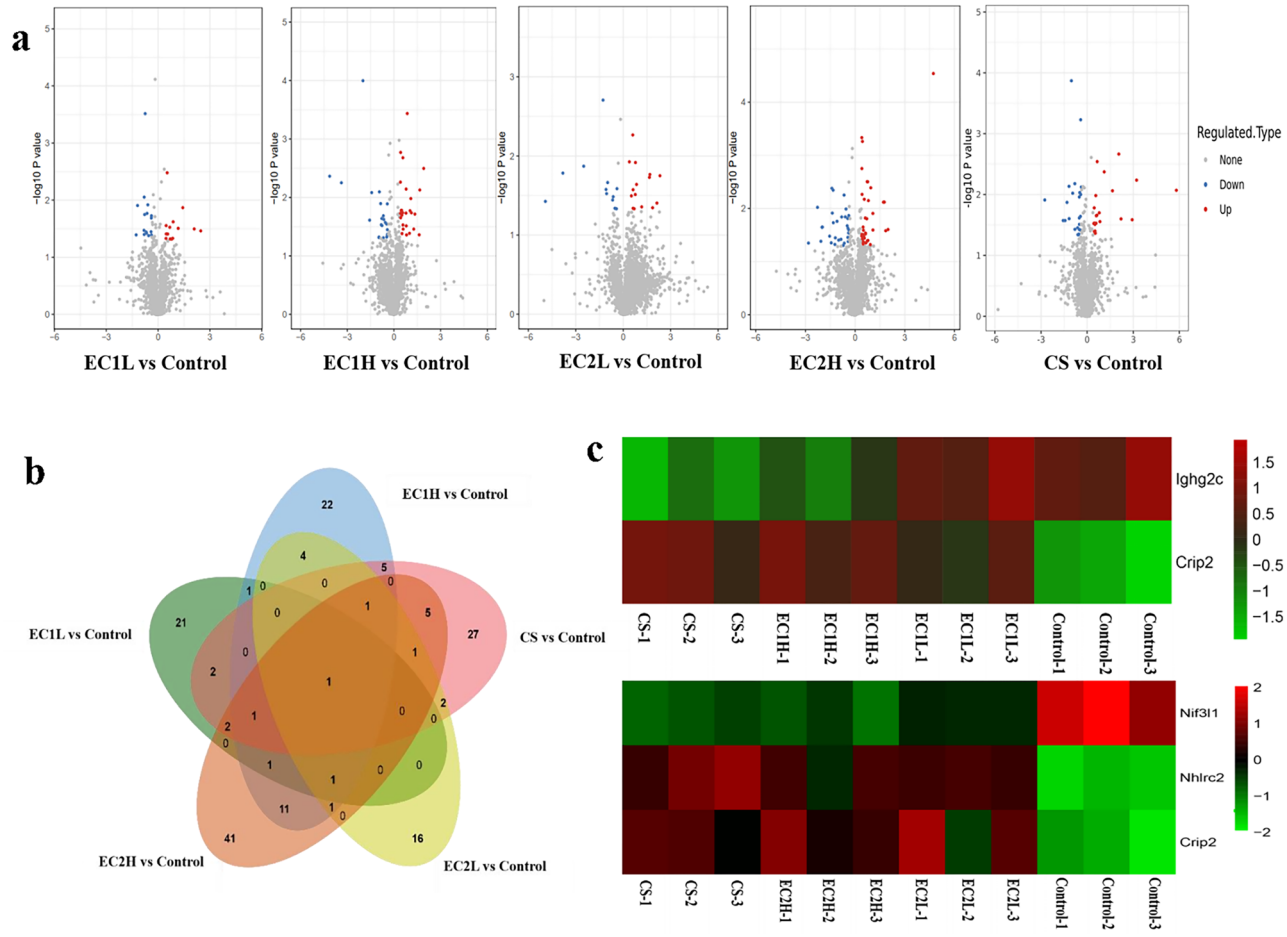
Analysis of differentially expressed proteins in cardiac tissue. (**a**) Volcano plot was drawn with the fold change transformed by Log2 as the horizontal axis and the logarithm of the P-value (− Log10) as the vertical axis. The red dot represents the up-regulated proteins, and the blue dot represents the down-regulated proteins. (**b**) The approximate relationship between differentially expressed proteins in EC and CS was plotted using a Venn diagram. (**c**) Heatmap showing the expression of common differentially expressed proteins, with red indicating up-regulation and green indicating down-regulation.

After 10 weeks of exposure, the cardiac tissues were analyzed by proteomics. According to the criteria of fold change (FC) > 1.3 and *P* value < 0.05, the differentially expressed proteins were screened. Among them, the expression of 14 proteins were up-regulated and 16 proteins were down-regulated in the EC1L group, 26 proteins were up-regulated and 23 proteins were down-regulated in the EC1H group, 15 proteins were up-regulated and 12 proteins were down-regulated in the EC2L group, 36 proteins were up-regulated and 30 proteins were down-regulated in the EC2H group, and 22 proteins were up-regulated and 25 proteins were down-regulated in the CS group (Fig. 2a). Venn diagrams (Fig. 2b) showed that only one protein cysteine-rich protein 2 (Crip2) overlapped in all the EC and CS groups. The heat map (Fig. 2c) showed that the common differentially expressed proteins were mainly immunoglobulin heavy constant gamma 2C (Ighg2c) and Crip2 between EC1 and CS, and NGG1 interacting factor 3-like 1 (Nif3l1), NHL repeat-containing protein 2 (Nhlrc2) and Crip2 between EC2 and CS. Though the expression of Ighg2c and Nif3l1 were down-regulated, and Nhlrc2 and Crip2 were up-regulated in all the sample groups, while the trend of up-regulated or down-regulated expression was more obvious in CS group, although with no significant difference.

**Figure 3:**
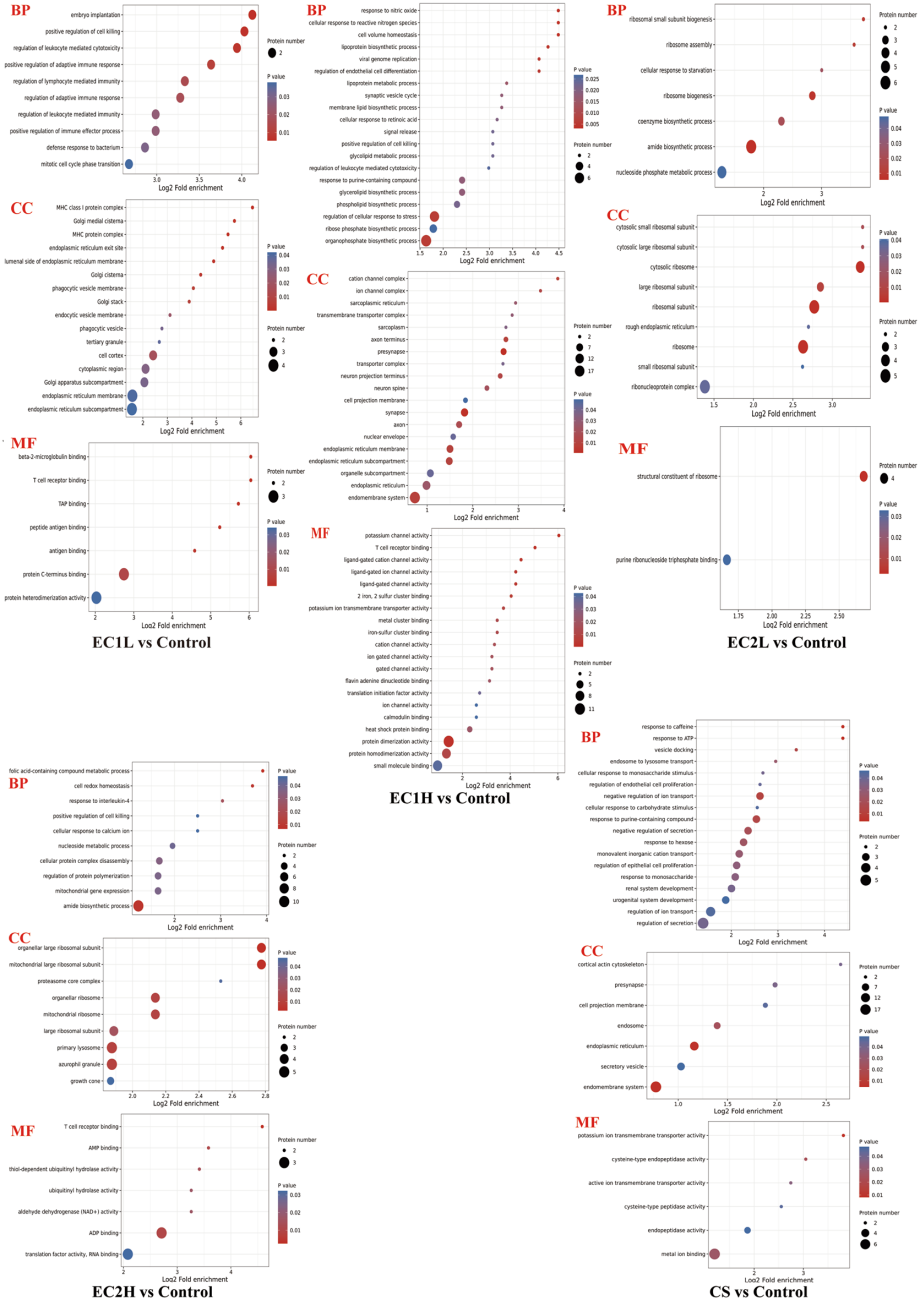
GO enrichment analysis of EC and CS groups vs. Control. The abscissa is the enrichment multiple, the ordinate is the GO entry, the circle color represents the enrichment significance (P value), and the circle size represents the number of differential proteins in the functional class.

Gene Ontology (GO) applies statistical analysis to establish the relationship between genes and their expressed proteins. Based on the eggNOG database, GO was analyzed by using eggnog mapper software (v2.0) to enrich the biological process (BP), cellular component (CC), and molecular function (MF) of differentially expressed proteins. As shown in the bubble diagram (Fig. 3), in terms of BP, the differential proteins mainly focused on embryo implantation, positive regulation of cell killing and regulation of leukocyte mediated cytotoxicity in EC1L group, focused on cell volume homeostasis, response to nitric oxide and cell response to reactive nitrogen in EC1H group, focused on amide biosynthesis, ribosome biogenesis and ribosome small subunit biogenesis in EC2L group, and focused on folic acid containing compound metabolism, amide biosynthesis and cell redox homeostasis in EC2H group, respectively. In comparison, the differential proteins in CS group mainly focused on the negative regulation of the reaction to caffeine, adenosine-triphosphate (ATP) and ion transport. In terms of MF, the differential proteins were mainly focused on β 2-microglobulin, T-cell receptor and tumor abnormal protein (TAP) bind in EC1L group, on potassium channels, protein dimer activity, and T-cell receptor binding in EC1H group, on the structural components of ribosome and combines with purine ribonucleoside triphosphate in EC2L group, and on T cell receptor binding, adenosine diphosphate (ADP) binding and adenosine monophosphate (AMP) binding in EC2H group, respectively. In contrast, the differential proteins were mainly focused on potassium transmembrane transporter activity, cysteine endopeptidase activity and metal ion binding in CS group. In conclusion, the differential proteins were enriched in T-cell receptor binding in EC1L and EC1H groups, and enriched in the biosynthesis of large ribosomal subunits and amides in EC2L and EC2H groups.

**Figure 4:**
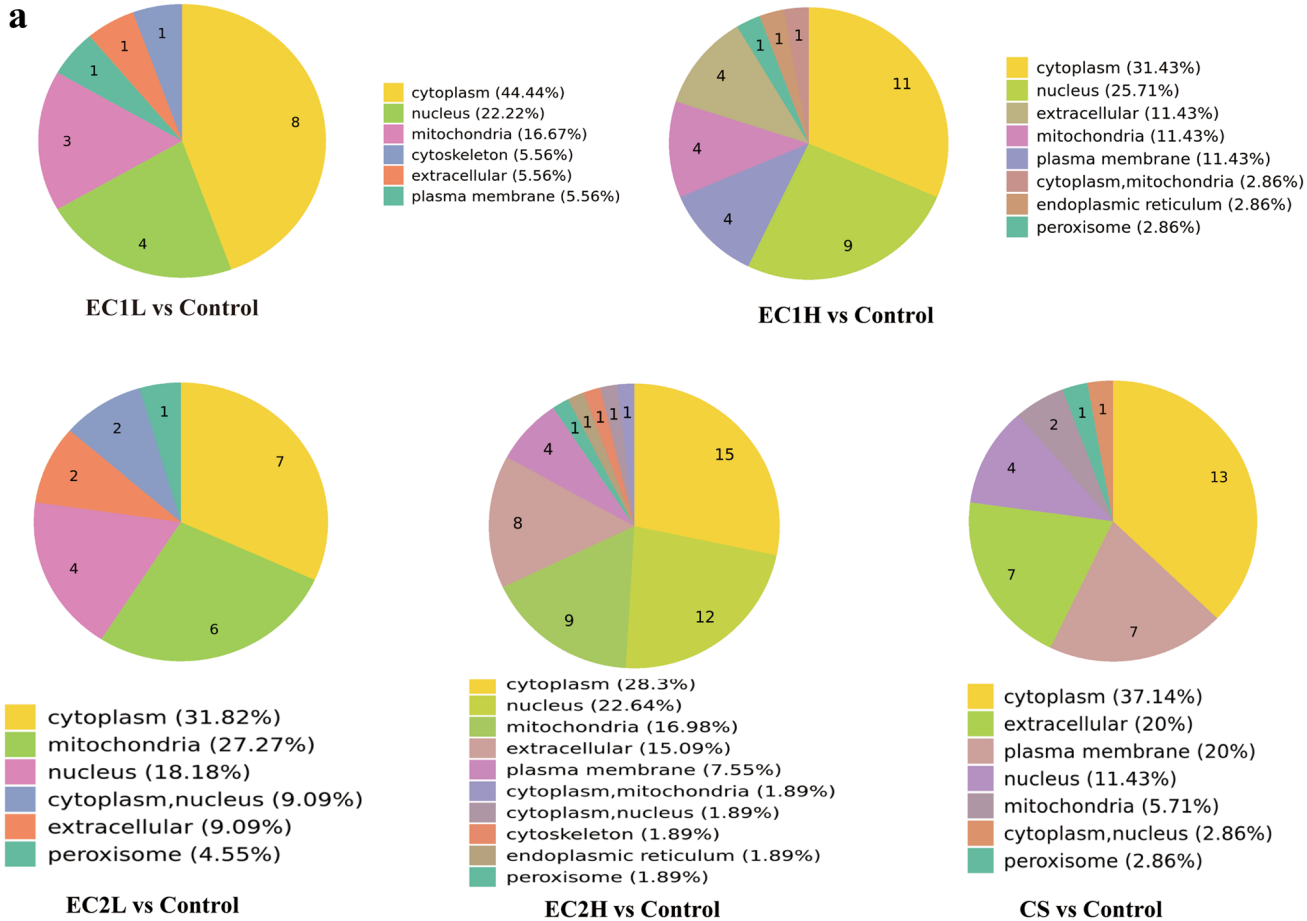

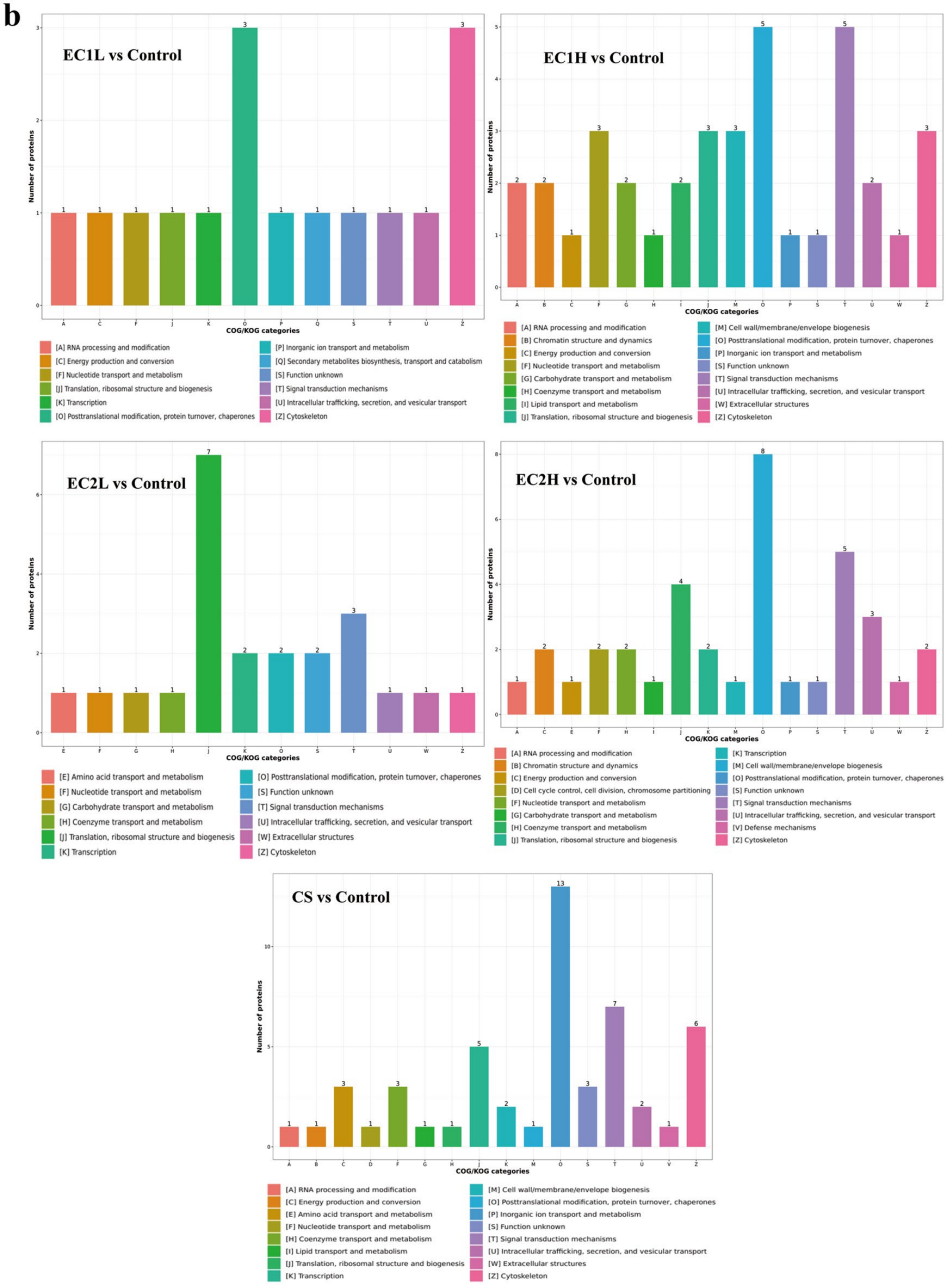
Enrichment analysis of differentially expressed proteins. (**a**) Differential proteins in each group were predicted for subcellular localization using Wolfpsort software. The numbers in the fan chart represent the number of differential proteins, and different colors mark the classification of subcellular structure and the percentage of differential proteins. (**b**) The COG/KOG functional classification of differentially expressed proteins was performed by database matching analysis. The abscissa is the COG/KOG functional classification, and the ordinate is the protein quantity.

Subcellular structural localization prediction and classification statistics of differentially expressed proteins were also carried out. As shown in Fig. 4a, the differentially expressed proteins were mostly distributed in the cytoplasm and nucleus in EC1 groups, in the cytoplasm, mitochondria and nucleus in EC2 groups, and in the cytoplasm, extracellular and plasma membrane in CS group. COG annotation extracts the GO items annotated by the separated and identified differential proteins separately and draws them into a column chart, which can predict the functional classification of the differential proteins. For both EC and CS, most of the proteins were involved in signal transduction mechanisms, posttranslational modification, protein conversion and chaperone function (Fig. 4b).

### Toxic effects of EC and CS on the gastrointestinal system in mice

**Figure 5:**
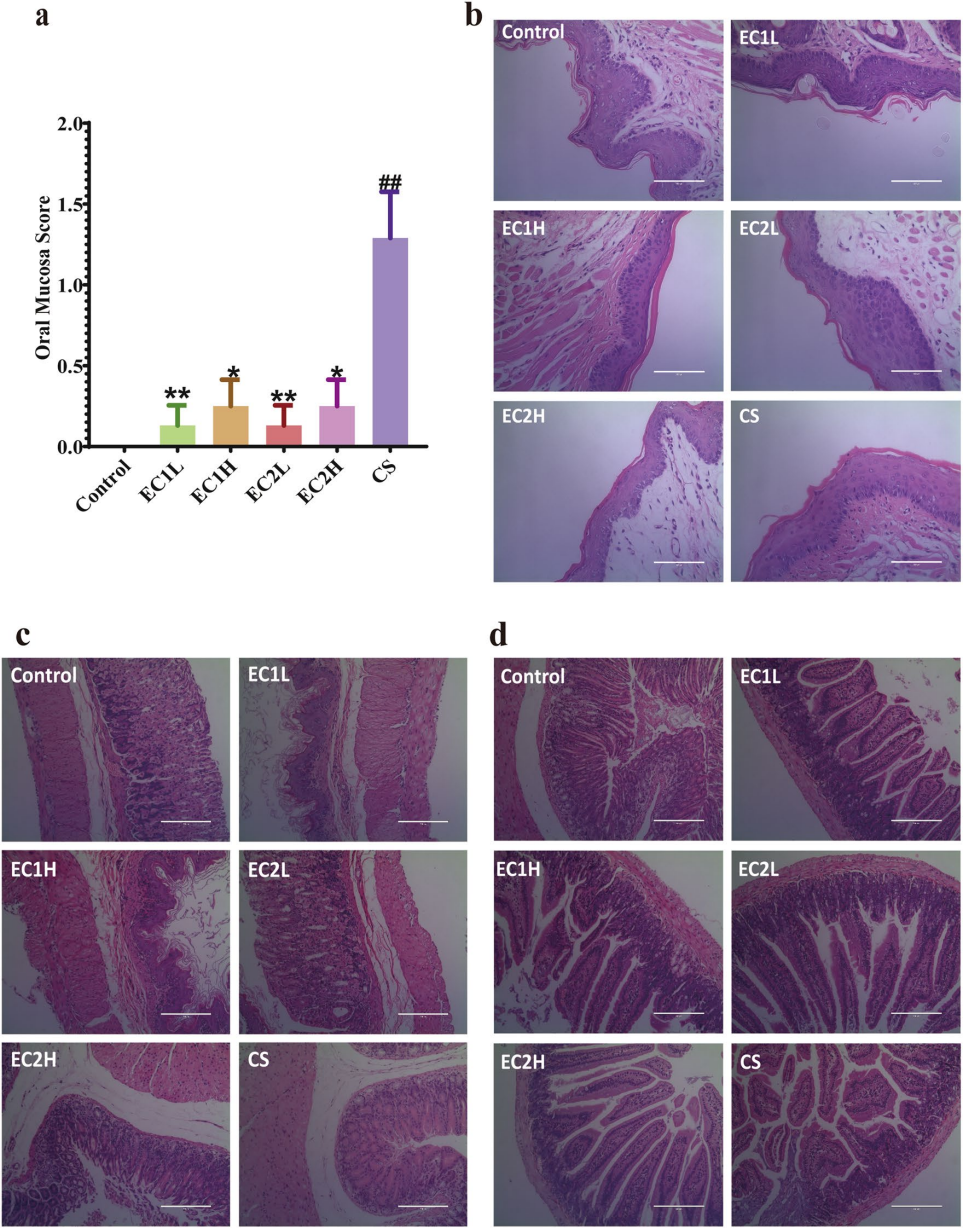
Effects of EC and CS on (**a**) oral mucosa score (n = 7–8); pathological changes of (**b**) oral mucosa (HE, × 400), (**c**) gastric (HE, × 200) and (**d**) duodenal (HE, × 200) in mice. Data are represented as the mean ± SEM. ^#^*P* < 0.05, ^##^*P* < 0.01 vs. Control; *P < 0.05, **P < 0.01 vs. CS.

According to the Sonis standard, the oral mucosa of animals was scored (Fig. 5a). Except for the control group, the oral mucosa of mice in the sample groups were abnormal, and the score increased. Compared with the control group, the CS group had statistical difference (*P* < 0.01). The oral mucosa scores in the EC groups were significantly lower than those in the CS group (*P* < 0.01 or *P* < 0.05). Histopathological changes in the oral mucosa were observed by HE staining (Fig. 5b). The epithelial structure of the oral mucosa in the control group was basically normal, the cells were arranged in an orderly manner, and there was no telangiectasia. In the EC groups, the integrity of the oral mucosal epithelium was slightly poor, thinning occasionally, and there was no obvious telangiectasia and congestion. The results also showed that the integrity of oral mucosal epithelium in the CS group was poor, the nail mutation was flat, and there was no obvious capillary dilatation or congestion. These results indicated that smoking stimulated the oral mucosa of mice, and the damage to the oral mucosa caused by CS was greater than that caused by EC. HE staining was used to observe the gastric tissue (Fig. 5c).The gastric mucosa epithelium of each group was basically intact, with good continuity and orderly arrangement of glands. The mucosal tissue structure was clear, and no significant difference was found. For the duodenum (Fig. 5d), the intestinal villi of the normal control mice were basically neat and intact, the central chylous duct was clearly visible, and the glandular structure was normal. In the EC groups, slight edema and short villi were occasionally observed. Mild to moderate edema and shortening of villi were observed in the CS group. There were no significant differences among all the groups. HE staining results (Suppl. Fig. S3a) showed that the liver cells of mice in each group were radially distributed, closely structured, and neatly arranged, with clear outlines and stained cytoplasm. No obvious abnormal pathological changes were observed in each group. Masson staining results (Suppl. Fig. S3b) showed that a few fibrous septa were formed in the liver tissues of mice in each group, the lobule structure was complete, and collagen fiber deposition was not significantly different among all the groups. There was an increasing trend in liver index among the EC and CS groups, but there was no significant difference (Suppl. Fig. S3c).

### The effects of EC and CS on the mouse kidney

**Figure 6:**
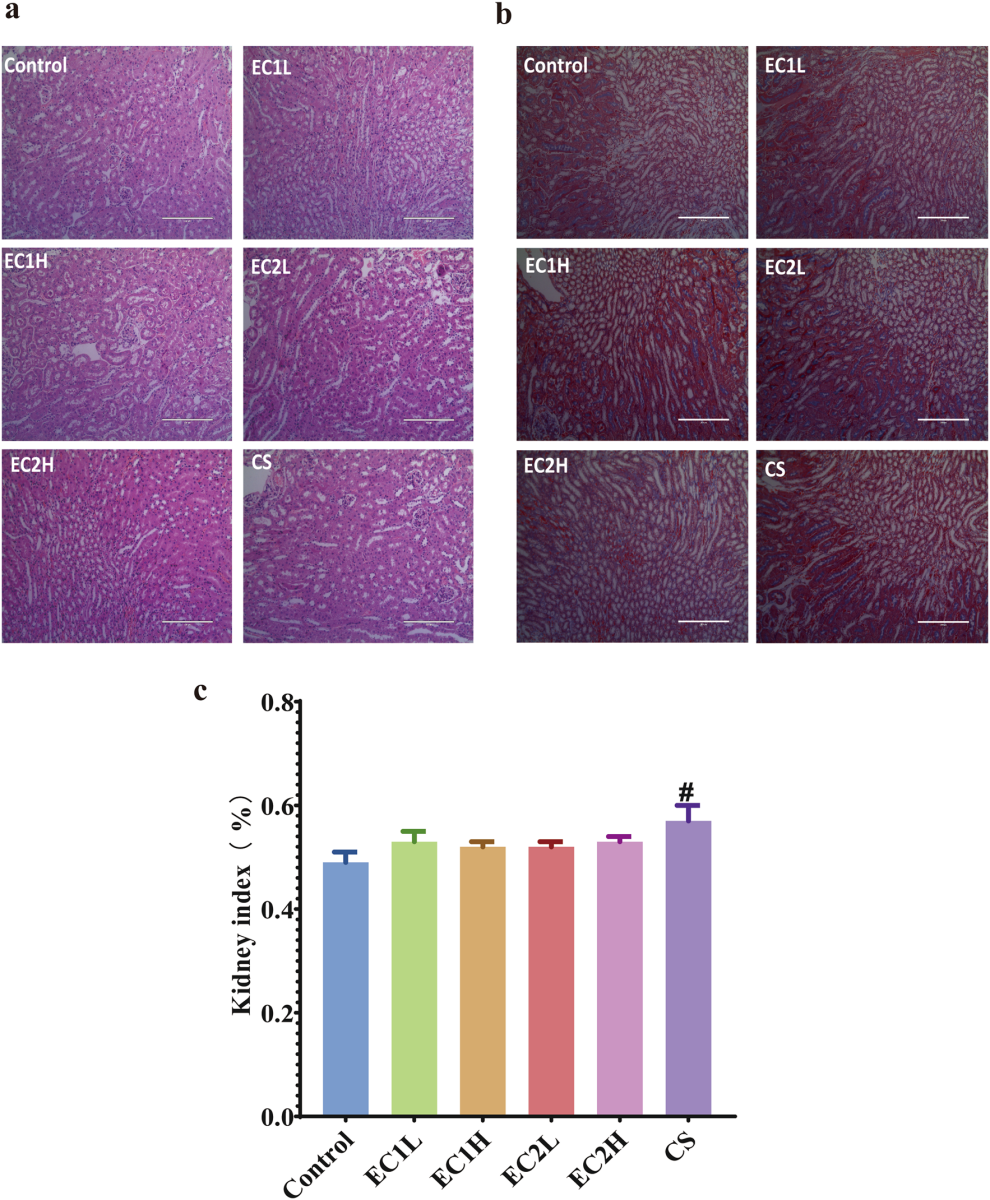
Pathological changes in the mouse kidney. (**a**) HE, × 200; (**b**) Masson, × 200; (**c**) kidney index (n = 7–8). Data are represented as the mean ± SEM. ^#^*P* < 0.05 vs. Control.

The results of HE staining (Fig. 6a) showed that the glomerular structure was intact, the size and morphology of renal tubules were not abnormal, the morphology and structure of renal tissues were not abnormal, and there was no obvious inflammatory cell infiltration. Masson staining showed that there was no significant difference in blue staining collagen fiber tissue and no obvious abnormality in glomeruli and renal vesicles (Fig. 6b**).** The kidney index (Fig. 6c) showed that compared with the control group, the kidney index of the CS group had an increasing trend, and the CS group was the most significant (*P* < 0.05), but there was no statistical difference in the other EC groups.

## Discussion

Smoking is a common and important risk factor for cardiovascular disease, which is the leading cause of premature death worldwide^37^. Blood oxygen saturation is an important characteristic reflecting the function of the respiratory system and circulatory system, as well as an important physiological parameter for early warning of cardiovascular disease^38^.

In this study, it was found that CS exposure significantly reduced the blood oxygen saturation of mice, and there were significant differences between the CS group and EC groups. On the one hand, CS produces carbon monoxide in the combustion process, which destroys the combination of oxygen and hemoglobin and affects the transportation and supply of oxygen. On the other hand, CS significantly reduced the ventilation function of the lung, and finally reduced blood oxygen saturation^39^. A study assessing the effects of e-cigarette use on atherosclerosis in smokers showed no changes in stiffness or reflexes, and no significant increases in systolic blood pressure, diastolic blood pressure, or heart rate^40^. Olfert et al.^41^ demonstrated that long-term exposure to EC, even at relatively low levels, could significantly increase arterial stiffness. Therefore, the potential role of the long-term effects of EC and CS in the development of cardiovascular disease should be further investigated.

We employed proteomics analysis to elucidate the mechanism of physiological and pathological changes in the cardiovascular system of mice under different smoke/aerosol exposure conditions. Previous studies have reported the effect of EC and CS on mouse lung samples by proteomics analysis^42^, but few reports have been made on the proteomics analysis of heart samples. Therefore, the effects of EC and CS on the heart by proteomics analysis of heart tissue in mice were studied here. In this study, among the common differentially expressed proteins, the expression trend of the EC groups were closer to that of the control group comparing with the CS group, and the CS group had a greater range of changes with more obvious biological effects. The differentially expressed proteins were mainly distributed in the cytoplasm and other places in the EC and CS groups. They exerted their biological effects by changing the signal transduction mechanism and biological functions such as posttranslational modification, protein conversion and chaperone function. Meanwhile, we found the common differential protein Crip2 (up-regulated expression) between EC and CS groups. Crip2 is a member of the LIM domain protein family, which belongs to the cysteine-rich intestine protein family l and contains between one and three LIM domains. Crip2 expression was detected in developing cardiac endothelial cells and adult hearts, and it was identified as a cardiovascular marker and plays an important role in regulating angiogenesis^43^. Therefore, Crip2 may mediate the effects of exposure on the heart and cardiovascular system by activating relevant signal pathways in mice exposed to EC and CS, and its specific mechanism needs further study.

In addition, both the EC and CS exposure caused a certain degree of damage to the gastrointestinal system of mice, and CS exhibited more severe damage to the oral cavity. This is consistent with previous research suggesting that CS and its chemical constituents may exacerbate ulcer formation^44^. Studies have found that smokers are almost twice as likely to develop peptic ulcers as nonsmokers^45^. According to clinical observations, smokers have a higher risk of gastrointestinal diseases and are more difficult to heal than nonsmokers^46,47^. Similarly, smoking affects the protective mechanisms of the gastroduodenal mucosa, increases the risk of Helicobacter pylori infection, and can cause harmful duodenal contents to return to the stomach, adversely affecting the gastrointestinal system^48^.

Liver diseases are generally considered to be related to obesity and alcohol consumption. Although the inhaled tobacco substances are first metabolized in the liver after smoking, hepatologists have traditionally paid little attention to smoking related diseases^49^. Approximately 40% of patients with liver disease have a history of smoking. Clinical evidence shows that smoking has a negative impact on the incidence rate of fatty liver, the progression of liver fibrosis, the development of liver cancer and the prognosis of patients with advanced liver disease^50^. The subchronic toxic effects of EC and CS exposure on the liver were studied here. Although there was no significant effect on the pathological changes in the mouse liver, smoking caused an increase in the liver index. The relationship between smoking and chronic liver disease deserves further assess. Many studies have shown that there is a relationship between smoking and chronic kidney disease. CS could accelerate oxidative stress and increase the production of reactive oxygen species to induce chronic kidney disease^51,52^. In this study, the effects of EC and CS exposure on the kidneys of mice has also been evaluated, and the effect of CS on the kidney index was greater than that of EC. Although there was no significant difference in the kidney damage caused by EC exposure under different nicotine concentrations, it seems necessary to observe the mechanism of kidney toxicity caused by smoking by further extending the exposure time.

Nicotine is the main ingredient in smoking products and can be absorbed through a variety of pathways, including the oral mucosa, lungs, skin or intestines^53^. The nicotine concentrations varies from 0 to 36.6 mg/mL in most e-liquids^54^. Unfortunately, the research on the nicotine effects of EC is limited and controversial. Studies confirm that the cardiovascular effects of nicotine appear to depend on dose and its distribution dynamics^55,56^. The high-dose and low-dose groups designed in this study showed no significant difference after 10 weeks of exposure, and further dose changes may be needed to explore the effects of different doses of nicotine on the cardiovascular and gastrointestinal systems.

EC use was associated with milder pathological changes than CS use in this study, agreeing with previous studies that EC cannot be considered completely safe^57,58^. To meet smokers’ diverse needs, EC has developed into a variety of flavors. Currently, most studies evaluating the effects of EC on various biological systems have focused on nicotine in e-liquids, and neglected flavoring as a potential variable of toxicological concern. The flavor components in different EC aerosols are significantly different.

Although these ingredients are considered safe when ingested orally, there is still considerable uncertainty about the risks when ingested through inhalation. So the potential risks of cardiovascular and gastrointestinal effects of EC with different flavors needed to further evaluate. Some studies have shown that some flavoring agents in EC could induce cytotoxicity^59^, but there are also reports that flavoring has no significant effect on cytotoxicity and genotoxicity^60^. The preliminary testing in this study illustrated that no significant changes were observed between the two flavor ECs, which may have something to do with the chemical compositions of the flavours used in the e-liquid and requires further clarification.

However, the potential toxicity and long-term effects of EC and CS on the cardiovascular and gastrointestinal systems should be further investigated due to the limitation of experiment duration. Meanwhile, more clinical trials are needed to verify the outcome of the animal model and finally determine the safety and effectiveness of EC for human consumption.

## Conclusion

In this study, the potential effects of the cardiovascular, gastrointestinal and renal systems in mice were investigated after exposure on the aerosols of four e-cigarette groups with two different flavors and two nicotine doses together with CS. The results showed that both EC and CS reduced blood oxygen saturation, and CS seems to be more harmful. The differentially expressed protein Crip2 of EC and CS may mediate the effects of smoking on the heart and cardiovascular systems by activating relevant signal pathways, and its specific mechanism needs further study. In addition, both the EC and CS exposure caused certain damage to the gastrointestinal tract of mice, and CS exhibited more harmful to the oral cavity than EC. In all the groups, the damage to the stomach and duodenum was slight, and there was no obvious pathological change in the liver and kidney.

## Supplementary Information

Supplementary Information.

## Data Availability

All data generated or analyzed during this study are included in this published article [and its supplementary information files].
