# Supplementary material for: The toxic effects of electronic cigarette aerosol and cigarette smoke on cardiovascular, gastrointestinal and renal systems in mice
Source: Sci Rep. 2023 Jul 31;13:12366. doi: 10.1038/s41598-023-39201-7 (PMC10390502; doi:10.1038/s41598-023-39201-7)
Supplement: Supplementary file 1 — Supplementary Information. [file 41598_2023_39201_MOESM1_ESM.pdf]

Supplementary Material

The toxic effects of electronic cigarette aerosol and cigarette smoke on cardiovascular, gastrointestinal and renal systems in mice

Aijing Xu[1], Kun Duan[3], Wanchun Yang[1], Guodong Feng[1], Zehong Wu[3] ,Xingtao Jiang[3], Min Li[1, 2], Peiqing Liu[1, 2 *], Jianwen Chen[1, 2 *]

[1] Department of Pharmacology and Toxicology, School of Pharmaceutical Sciences, Sun Yat-Sen University, Guangzhou 510006, Guangdong, China.

[2] National and Local Joint Engineering Laboratory of Druggability and New Drugs Evaluation, Guangdong Engineering Laboratory of Druggability and New Drug Evaluation, School of Pharmaceutical Sciences, Sun Yat-sen University, Guangzhou 510006, China.

[3] RELX Science Center, Shenzhen RELX Tech. Co., Ltd., Shenzhen 518101, China.

[*] Corresponding author

E-mail addresses: chenjwen@mail.sysu.edu.cn; Tel: +86-020-3994-3062

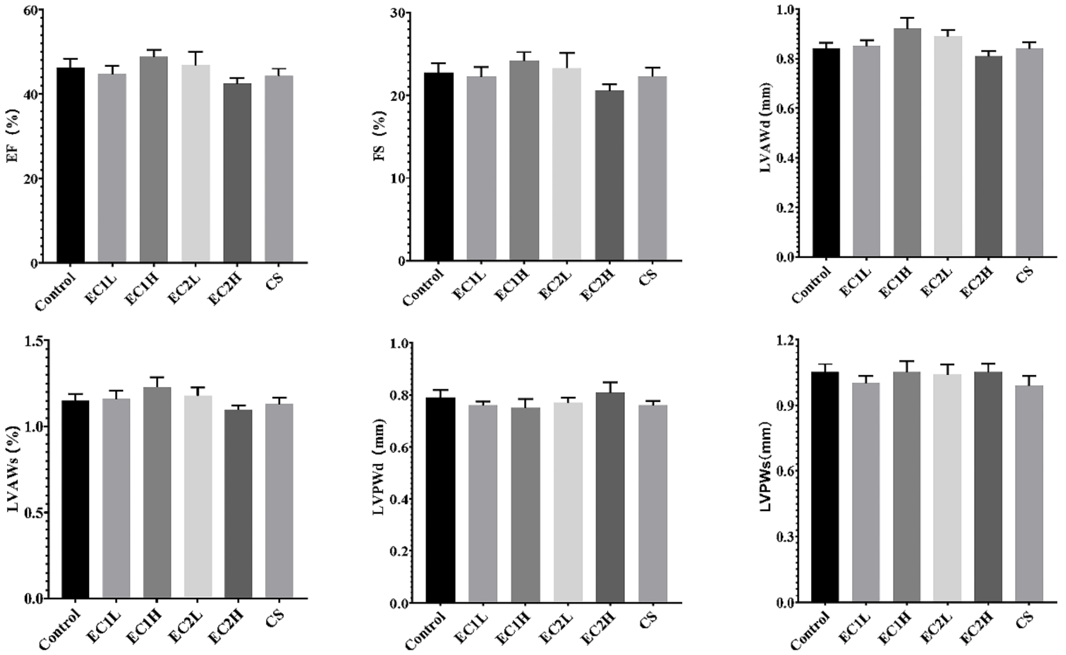

Figure S1. Effects of EC and CS on cardiac function in mice（n=7-8）. Data are represented as mean± SEM.

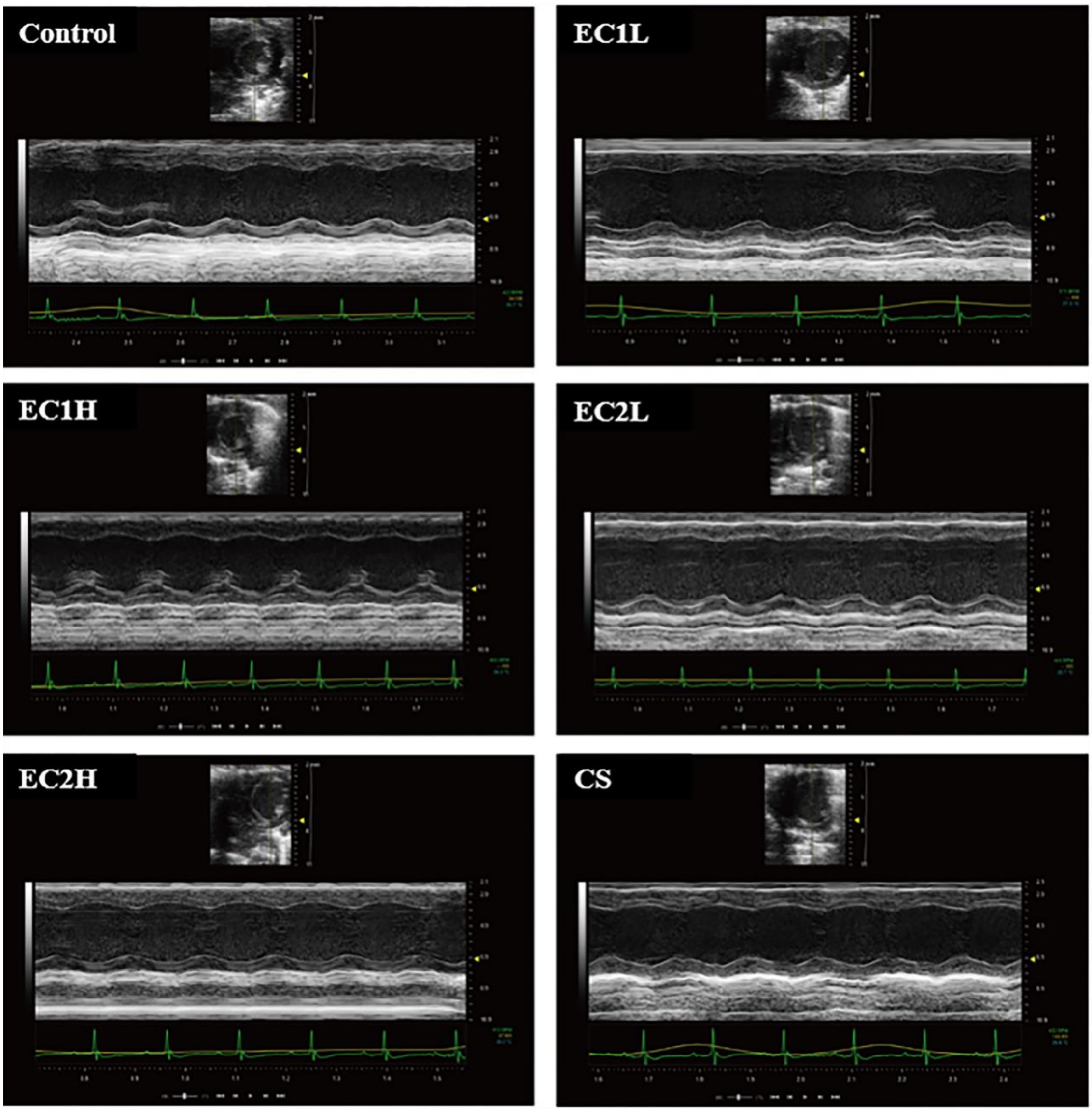

Figure S2. Ultra- high resolution ultrasound image of small animal heart.

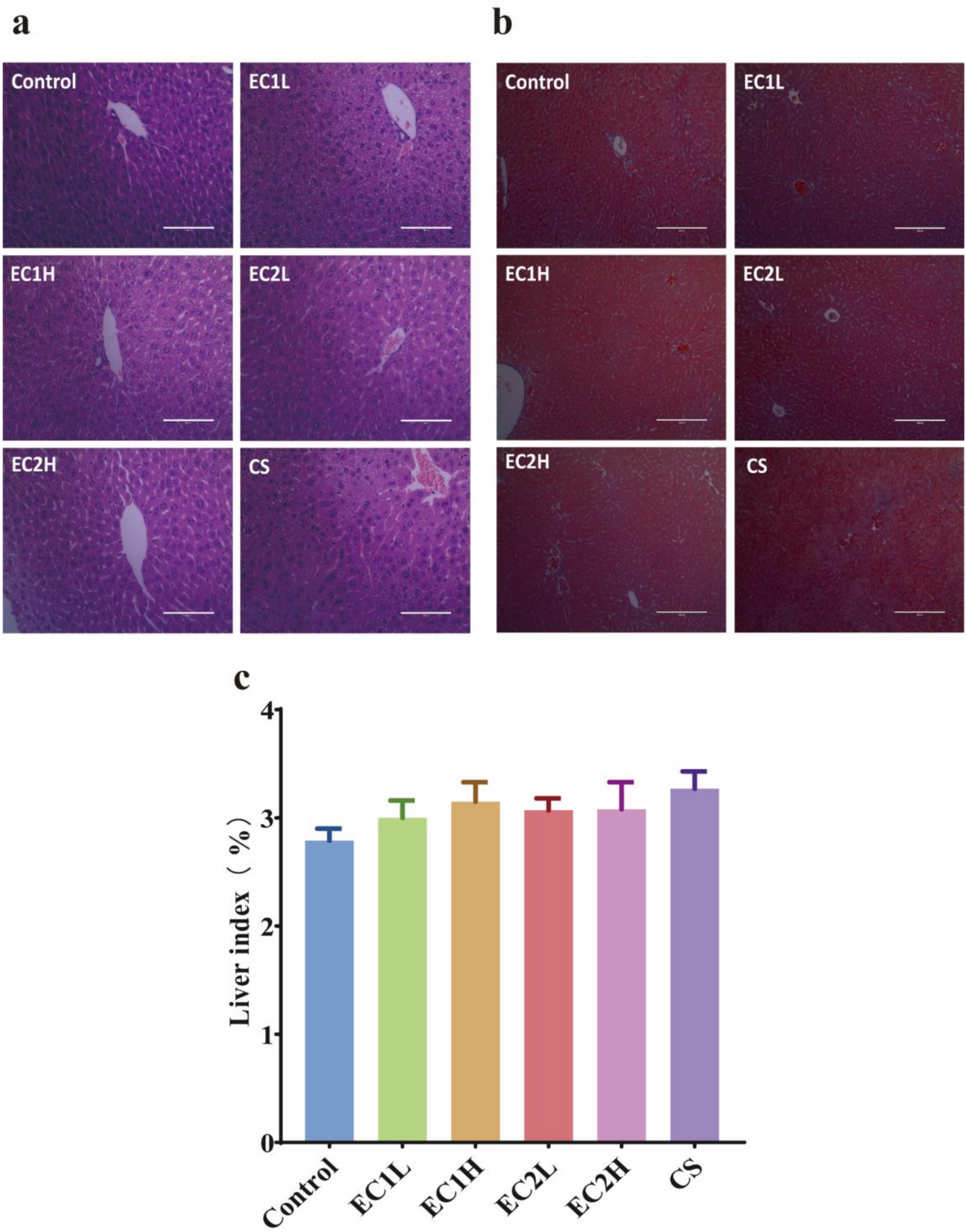

Figure S3. Pathological changes of liver in mice. (**a**) (HE, ×400) and (**b**) (Masson, ×200) (**c**) Liver index (n=7–8).

RAW data

Fig.1a

	Control	EC1L	EC1H	EC2L	EC2H	CS
	95.76	87.12	91.94	95.83	96.11	79.05
	96.26	90.56	85.86	93.06	90.26	84.17
	98.99	85.02	90.89	92.43	89.57	79.29
	90.6	90.73	96.38	94.56	93.51	80.41
	92.84	95.29	94.03	92.61	92.61	79.3
	95.23	93.73	91.02	98.49	91.87	78.21
	98.07	94.36	85.82	90.46	89.75	85.62
	98.06	91.43	89.87	87.76	84.29	
**mean**	**95.73**	**91.03**	**90.73**	**93.15**	**91.00**	**80.86**
**SEM**	**1.01**	**1.26**	**1.29**	**1.16**	**1.23**	**1.08**

Fig.1b

	Control	EC1L	EC1H	EC2L	EC2H	CS
	587.04	571.55	563.77	593.84	652.74	654.73
	633.56	636.61	606.49	596.70	518.67	580.31
	592.19	658.76	643.88	655.89	663.63	626.36
	599.54	637.68	624.32	575.47	587.56	607.01
	543.84	635.85	608.92	596.02	662.90	685.73
	573.96	548.00	658.86	604.48	583.79	635.22
	561.24	582.55	593.32	576.84	596.25	602.29
	619.11	587.99	612.11	533.25	622.73	
mean	**588.81**	**607.37**	**613.96**	**591.56**	**611.03**	**627.38**
SEM	**10.4**	**14.03**	**10.39**	**12.13**	**17.61**	**13.34**

Fig.1d

	Control	EC1L	EC1H	EC2L	EC2H	CS
	0.47	0.55	0.44	0.56	0.51	0.55
	0.52	0.66	0.58	0.57	0.52	0.61
	0.55	0.54	0.64	0.51	0.57	0.56
	0.56	0.45	0.55	0.59	0.47	0.55
	0.50	0.51	0.51	0.50	0.52	0.51
	0.47	0.57	0.54	0.52	0.54	0.55
	0.49	0.52	0.56	0.49	0.56	0.56
	0.52	0.52	0.53	0.51	0.54	
mean	**0.51**	**0.54**	**0.54**	**0.53**	**0.53**	**0.56**
SEM	**0.01**	**0.02**	**0.02**	**0.01**	**0.01**	**0.01**

Fig.5a

	Control	EC1L	EC1H	EC2L	EC2H	CS
	0	0	0	0	0	1
	0	0	0	0	1	2
	0	0	0	0	0	1
	0	0	1	0	0	2
	0	0	0	0	1	2
	0	0	1	0	0	1
	0	1	0	1	0	0
	0	0	0	0	0	
mean	**0.00**	**0.13**	**0.25**	**0.13**	**0.25**	**1.29**
SEM	**0**	**0.13**	**0.16**	**0.13**	**0.16**	**0.29**

Fig.6c

	Control	EC1L	EC1H	EC2L	EC2H	CS
	0.48	0.54	0.56	0.55	0.59	0.50
	0.50	0.47	0.53	0.54	0.51	0.50
	0.55	0.53	0.47	0.52	0.53	0.57
	0.56	0.50	0.54	0.54	0.53	0.58
	0.39	0.46	0.55	0.49	0.58	0.71
	0.55	0.62	0.46	0.56	0.49	0.59
	0.48	0.57	0.50	0.48	0.51	0.54
	0.44	0.52	0.54	0.52	0.54	
mean	**0.49**	**0.53**	**0.52**	**0.52**	**0.53**	**0.57**
SEM	**0.02**	**0.02**	**0.01**	**0.01**	**0.01**	**0.03**

Fig.S3 c

	Control	EC1L	EC1H	EC2L	EC2H	CS
	2.74	3.09	2.35	3.54	2.03	3.41
	2.86	2.62	2.77	3.19	2.68	3.97
	3.37	3.21	3.21	2.66	3.36	2.92
	2.88	2.68	3.46	3.41	3.29	2.87
	2.23	2.70	3.90	3.15	2.49	3.41
	2.94	3.97	3.31	2.83	4.37	3.46
	2.75	2.82	3.47	2.83	3.08	2.85
	2.58	2.88	2.72	2.94	3.33	
mean	**2.79**	**3.00**	**3.15**	**3.07**	**3.08**	**3.27**
SEM	**0.11**	**0.16**	**0.18**	**0.11**	**0.25**	**0.16**

Fig.S1

FS（%）	Control	EC1L	EC1H	EC2L	EC2H	CS
	15.96	19.83	22.31	25.23	21.69	20.95
	22.74	24.37	23.47	14.06	22.25	25.47
	21.76	22.43	26.58	26.77	19.66	25.26
	22.01	22.68	21.75	22.25	18.74	17.53
	25.33	16.60	23.57	20.41	23.08	23.29
	25.43	20.41	26.35	20.18	22.77	22.09
	27.07	27.31	20.74	30.41	18.14	21.51
	21.26	24.48	29.10	27.23	18.20	
mean	**22.70**	**22.26**	**24.23**	**23.32**	**20.57**	**22.30**
SEM	**1.21**	**1.17**	**1.01**	**1.82**	**0.74**	**1.04**

EF（%）	Control	EC1L	EC1H	EC2L	EC2H	CS
	34.01	40.93	45.80	50.34	43.74	43.29
	46.28	48.97	47.56	30.42	45.55	50.87
	46.42	45.86	53.03	52.84	41.03	42.16
	44.71	46.35	44.48	45.55	39.17	36.85
	50.43	35.35	47.50	41.88	46.77	44.61
	50.70	42.17	52.44	41.66	46.75	48.36
	53.63	53.55	42.80	58.62	38.17	44.02
	43.77	44.81	56.67	53.83	38.34	
mean	**46.24**	**44.75**	**48.79**	**46.89**	**42.44**	**44.31**
SEM	**2.11**	**1.93**	**1.69**	**3.16**	**1.31**	**1.7**

LVAW;d（mm）	Control	EC1L	EC1H	EC2L	EC2H	CS
	0.83	0.73	0.95	0.97	0.74	0.93
	0.96	0.91	1.01	0.81	0.82	0.90
	0.86	0.86	0.75	0.85	0.85	0.85
	0.85	0.93	0.74	0.94	0.88	0.70
	0.83	0.92	0.90	0.81	0.72	0.84
	0.81	0.82	0.89	0.89	0.86	0.84
	0.86	0.78	1.10	1.00	0.77	0.86
	0.72	0.86	1.04	0.84	0.85	
mean	**0.84**	**0.85**	**0.92**	**0.89**	**0.81**	**0.84**
SEM	**0.02**	**0.02**	**0.05**	**0.03**	**0.02**	**0.03**

LVAW;s（mm）	Control	EC1L	EC1H	EC2L	EC2H	CS
	1.18	1.05	1.23	1.33	1.11	1.25
	1.26	1.22	1.26	1.04	1.08	1.13
	1.15	1.17	1.03	1.16	1.17	1.11
	1.09	1.26	1.00	1.18	1.20	0.94
	1.15	1.26	1.34	1.03	1.07	1.16
	1.23	1.01	1.13	1.15	1.03	1.17
	1.23	0.97	1.40	1.42	1.02	1.18
	0.91	1.33	1.43	1.16	1.10	
mean	**1.15**	**1.16**	**1.23**	**1.18**	**1.10**	**1.13**
SEM	**0.04**	**0.05**	**0.06**	**0.05**	**0.02**	**0.04**

LVPW;d（mm）	Control	EC1L	EC1H	EC2L	EC2H	CS
	0.870833	0.7425	0.870833	0.7975	0.944167	0.7975
	0.7425	0.779167	0.715	0.760833	0.88	0.7975
	0.7896	0.753333	0.678333	0.806667	0.7975	0.7258
	0.7425	0.77	0.751667	0.7975	0.733333	0.678333
	0.715	0.7425	0.66	0.7425	0.678333	0.7402
	0.941875	0.7	0.733333	0.834167	0.953333	0.7947
	0.815833	0.843333	0.669167	0.733333	0.779167	0.788333
	0.715	0.751667	0.916667	0.669167	0.678333	
mean	**0.79**	**0.76**	**0.75**	**0.77**	**0.81**	**0.76**
SEM	**0.03**	**0.01**	**0.03**	**0.02**	**0.04**	**0.02**

LVPW;s（mm）	Control	EC1L	EC1H	EC2L	EC2H	CS
	0.980833	0.889167	1.310833	1.045	1.173333	1.081667
	1.081667	1.045	0.935	0.788333	1.164167	1.035833
	1.05216	1.0015	0.9625	1.2375	1.026667	0.933333
	0.925833	0.971667	1.081667	1.109167	0.916667	0.843333
	1.026667	1.118333	0.999167	1.054167	0.9625	0.8521
	1.189375	0.860417	0.980833	0.980833	1.173333	1.1508
	1.191667	1.1275	0.925833	1.1	1.045	1.054167
	0.916667	0.99	1.21	1.035833	0.9075	
mean	**1.05**	**1.00**	**1.05**	**1.04**	**1.05**	**0.99**
SEM	**0.04**	**0.03**	**0.05**	**0.05**	**0.04**	**0.04**
